# Effectiveness of a FODMAP-Based, Coach-Supervised, and Peer-Supported Lifestyle Program in Reducing Gastrointestinal and Extraintestinal Symptoms in Irritable Bowel Syndrome

**DOI:** 10.1016/j.gastha.2026.101087

**Published:** 2026-08-07

**Authors:** Facundo Pereyra, Francisco Schlottmann, Carolina Salvatori, Astrid Smolarczuk, Sofía Barbagelata

**Affiliations:** 1 Department of Gastroenterology, Cipolletti Hospital, Cipolletti, Río Negro, Argentina; 2 Department of Surgery, Hospital Alemán of Buenos Aires, Buenos Aires, Argentina; 3 Department of Gastroenterology, Hospital Alemán of Buenos Aires, Buenos Aires, Argentina

**Keywords:** Irritable Bowel Syndrome, Holistic Intervention, Diet, Exercise

## Abstract

**Background and Aims:**

We aimed to evaluate the impact of a comprehensive lifestyle program that integrates dietary, behavioral, and peer support strategies on gastrointestinal and extraintestinal symptoms in patients with irritable bowel syndrome (IBS).

**Methods:**

We performed a cross-sectional study analyzing a consecutive series of patients with IBS according to Rome IV criteria who participated in a structured, 3-week digital program between July 2022 and January 2025. The intervention combined a low-FODMAP, low-starch, low-sucrose diet with continuous guidance from a nutritionist-coach, daily educational materials (recipes, meal planning tips), peer support through shared group participation, and non-pharmacological tools such as mindfulness and physical activity. Symptom severity was assessed before and after the program using the IBS Severity Scoring System, the Patient Health Questionnaire-9 for depression, and Likert scales for extraintestinal symptoms.

**Results:**

A total of 14,186 patients with IBS were analyzed; the mean age was 44.5 (16–86) years, and 13,323 (93.9%) were female. IBS-M (mixed bowel habits) was the more frequent IBS subtype (n = 6,496, 45.8%); 8729 (61.5%) had ≥2 extraintestinal symptoms, and 6923 (48.8%) reported moderate or severe depressive symptoms. IBS-Severity Scoring System scores significantly decreased from 287.3 ± 69.5 to 188.3 ± 87.6 (*P* < .001), and Patient Health Questionnaire-9 scores improved from 10.2 ± 5.4 to 3.6 ± 3.4 (*P* < .001). All extraintestinal symptom scores showed significant reductions (*P* < .001), and 5540/8729 (63.5%) and 8403/11,746 (71.5%) reported >50% reduction of intensity in at least 2 extraintestinal digestive and non-digestive symptoms, respectively.

**Conclusion:**

A structured multidisciplinary intervention with dietary guidance and non-pharmacological strategies significantly improved both gastrointestinal and extraintestinal symptoms of IBS. A holistic approach addressing patients’ diet, exercise habits, well-being, and mental health is recommended for the management of this complex disease.

## Introduction

Irritable bowel syndrome (IBS) is a prevalent functional gastrointestinal disorder characterized by abdominal pain and altered bowel habits that can substantially reduce patients' quality of life and increase social costs.^1^^,^^2^ The global prevalence of IBS is approximately 14%, though this rate varies significantly across different countries (first-world countries have the highest prevalence).^3^

While the precise causes of IBS remain unclear, evidence suggests that disruptions in the gut-brain axis are involved in its development.^4^ Additionally, a persistent low-grade inflammatory response in the intestinal mucosa appears to contribute to symptom onset by increasing sensitivity in visceral and somatic areas.^5^ As a result, patients affected by IBS often suffer from other co-occurring diseases characterized by hypersensitivity including extraintestinal digestive (gastroesophageal reflux [GERD] or dyspepsia) and non-intestinal (fibromyalgia, asthma, chronic fatigue, migraine, and/or chronic pain syndromes among others) diseases.^6^, ^7^, ^8^

Dietary interventions are key components in the management of IBS.^9^ The low-FODMAP (fermentable oligo-, di-, monosaccharides, and polyols) diet has shown the most robust data for improving overall symptom burden.^10^, ^11^, ^12^ In fact, the American College of Gastroenterology guidelines recommend a limited trial of the low-FODMAP diet to improve global IBS symptoms.^13^ However, a large proportion of patients fail to achieve sustained symptom relief, often due to low adherence to such a restrictive diet.^14^ In addition, current treatment approaches mainly target abdominal symptoms and rarely address extraintestinal symptoms.^15^

We aimed to determine the impact of a comprehensive lifestyle program that integrates dietary, behavioral, and peer support strategies on gastrointestinal and extraintestinal symptoms in a large cohort of patients with IBS.

## Methods

### Study Design

We performed a cross-sectional study analyzing a consecutive series of patients undertaking the B15 program between July 2022 and January 2025. This is a social media–based program targeted for patients with functional gastrointestinal disorders that offers evidence-based recommendations to improve both gastrointestinal and extraintestinal symptoms. An electronic informed consent was obtained from all participants prior to data collection.

### Intervention

We conducted a digitally delivered 21-day multidisciplinary lifestyle intervention designed to improve gastrointestinal and extraintestinal symptoms in adults with functional digestive disorders. The program was structured around a comprehensive approach integrating dietary, physical, psychological, and social support strategies through a mobile platform.

The core of the intervention was a structured dietary protocol based on a temporary restriction of FODMAPs, along with reduced intake of sucrose and starch. All participants followed a standardized plan with a strict phase lasting 10 days, during which food intake was limited to a predefined list of tolerated items. The plan included recipes, shopping guides, and educational content to facilitate adherence. Although not individualized, adjustments were allowed based on pre-existing health conditions or emerging symptoms.

After the initial strict phase, participants initiated a guided reintroduction of food groups over the remaining days of the program. This stepwise process allowed identification of individual food triggers, under close monitoring by health coaches. Throughout the 21 days, participants were organized into peer groups and engaged in daily virtual sessions moderated by trained coaches. These meetings promoted adherence, provided emotional support, and offered a space to clarify doubts and share experiences. Adherence was assessed indirectly through daily check-ins, food logs, and participation in group discussions, allowing real-time feedback and adjustments.

**Figure 1:**
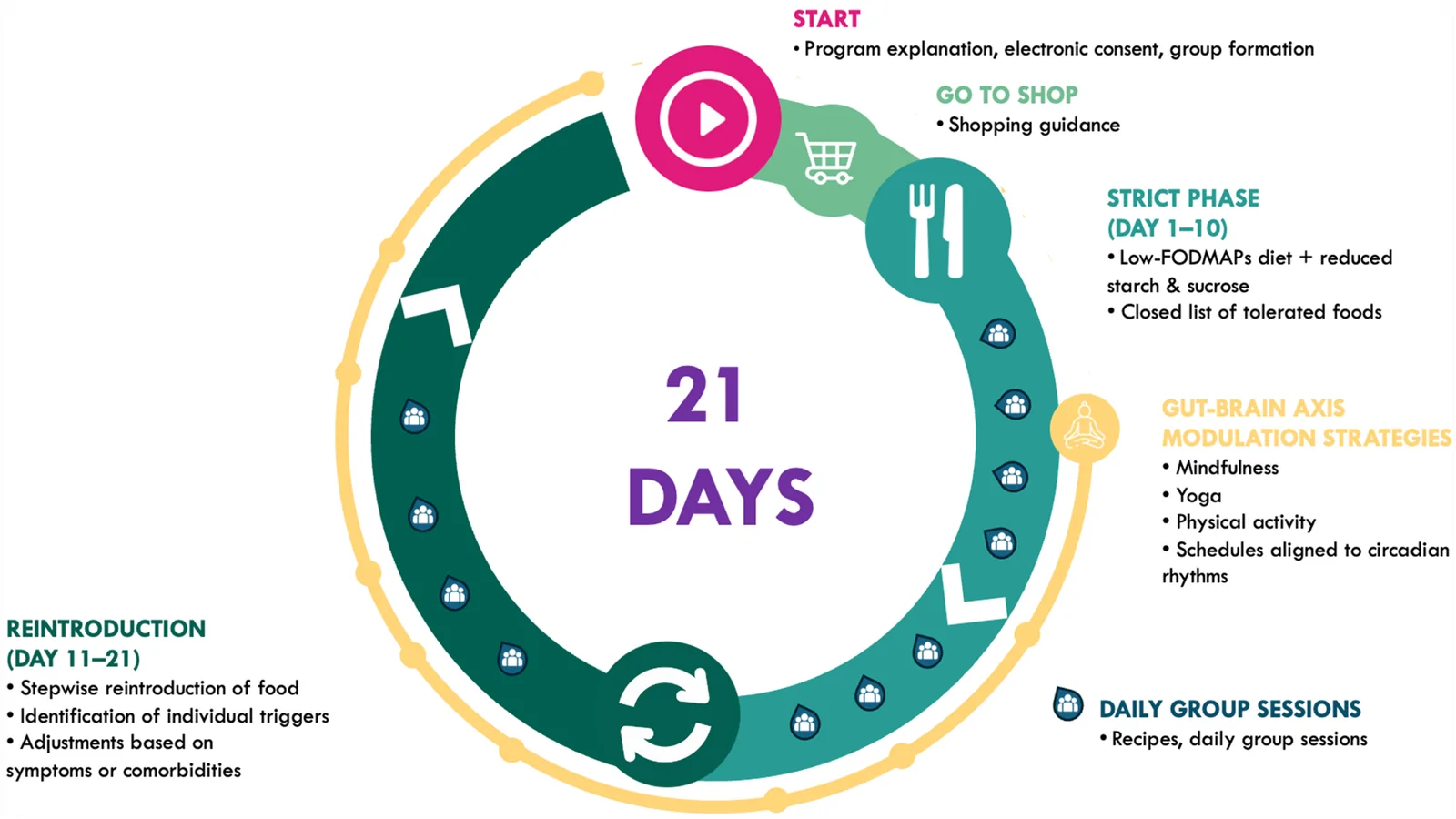
Visual representation of the 21-day digital intervention program for patients with functional gastrointestinal disorders. The circular timeline outlines the key phases of the program.

Beyond the dietary component, the program included scheduled mindfulness and physical activity sessions, such as yoga and low-impact workouts, offered online. These modules aimed to reduce stress, improve gut-brain axis regulation, and enhance physical and psychological well-being. Additionally, the daily schedule and meal timing were aligned with circadian rhythm principles, encouraging consistent routines to support digestion, metabolic health, and sleep quality. Participants experiencing moderate to severe or persistent symptoms during the intervention were referred to a gastroenterologist via telemedicine for evaluation and potential clinical management (Figure 1).

A structured online survey was administered at baseline and at the end of the 21-day program. The questionnaire, pilot-tested for clarity and relevance, included validated symptom assessment tools.

• For gastrointestinal IBS-related symptoms: Irritable Bowel Syndrome Severity Scoring System (IBS-SSS).^16^
• For mood symptoms: Patient Health Questionnaire-9 (PHQ-9).^17^
• For extraintestinal digestive and non-digestive symptoms: Likert-type scales assessing intensity of symptoms

Only adult participants (>18 years) with diagnosis of IBS (ie, Rome IV criteria) and who completed the full 21-day intervention and both pre- and post-program assessments were included in the final analysis. IBS was defined according to Rome IV criteria and classified according to the cathartic pattern into 3 groups: IBS-D (diarrhea-predominant IBS), IBS-C (constipation-predominant IBS), or IBS-M (mixed defecation pattern). Patients with overlapping intestinal conditions such as inflammatory bowel disease or celiac disease were excluded from the study.

### Variables and Outcomes

The following variables were extracted from all patients: age, gender, body mass index, IBS subtype, diabetes mellitus, hypothyroidism, food intolerance perception, history of cholecystectomy, and history of appendectomy. Extraintestinal digestive (GERD, dyspepsia, and belches) and depressive symptoms were assessed. The following extraintestinal non-digestive conditions associated with IBS were also queried: migraine, arthralgia, chronic fatigue, restless legs syndrome, extremity numbness, fibromyalgia, interstitial cystitis, plantar fasciitis, premenstrual syndrome, asthma, atopic dermatitis, recurrent candidiasis, and chronic cervicalgia.

The main outcome of the study was to determine clinical improvement of IBS-related symptoms and extraintestinal symptoms. Clinical improvements of IBS, depression, and extraintestinal symptoms were measured by the IBS-SSS, PHQ-9, and Likert-type scales assessing intensity after the intervention, respectively.

For IBS-related symptoms, a clinically meaningful improvement was defined as a decrease of 50 points or more in the total IBS-SSS score or a final score <150 points. For extraintestinal digestive and non-digestive symptoms, a reduction of >50% of symptom intensity in at least 2 symptoms was considered clinically relevant.

### Statistical Analysis

Categorical variables were described as percentages and numerical variables as means with their standard deviation. The chi-square test and the Mann-Whitney or Student *t*-tests were used to compare categorical and numerical variables between groups, respectively. The statistical analysis was performed using R (version 4.0.4) and R Studio (version 1.4.1106) software, with a *P* value <.05 considered statistically significant for all analyses.

## Results

A total of 14,186 patients with IBS were analyzed; the mean age of patients was 44.5 (16–86) years, and 13,323 (93.9%) were female. The mean body mass index was 26.2 (14–68) kg/m^2^. IBS-M was the more frequent IBS subtype (45.8%), followed by IBS-C (38.3%) and IBS-D (15.9%). Mild and moderate-severe depressive symptoms were reported in 37.8% and 48.8% of patients, respectively. The presence of at least 2 extraintestinal digestive and non-digestive symptoms was described by 8729 (61.5%) and 11,746 (82.8%) patients, respectively. The most frequent extraintestinal non-digestive symptoms were chronic cervicalgia (81.0%), chronic fatigue (67.5%), extremity numbness (60.2%), arthralgias (59.1%), and migraine (50.7%) (Table 1).

**Table 1:** Characteristics of Patients With Irritable Bowel Syndrome (IBS) Undertaking the Dietary Program

	N = 14,186
Mean age, y (range)	44.5 (16–86)
Female gender (%)	13,323 (93.9)
Mean BMI, kg/m^2^ (range)	26.2 (14–68)
Diabetes (%)	145 (1.0)
Hypothyroidism (%)	2114 (14.9)
Food intolerance perception (%)	890 (6.3)
Cholecystectomy (%)	1488 (10.5)
Appendectomy (%)	1393 (9.8)
Type of IBS	
IBS-D	2267 (15.9)
IBS-C	5423 (38.3)
IBS-M	6496 (45.8)
Extraintestinal digestive symptoms	
GERD (%)	7954 (56.1)
Dyspepsia (%)	8966 (63.2)
Belches (%)	8406 (59.3)
Depressive symptoms	
No (%)	1907 (13.4)
Mild (%)	5356 (37.8)
Moderate-severe (%)	6923 (48.8)
Extraintestinal symptoms	
Migraine (%)	7203 (50.7)
Arthralgia (%)	8387 (59.1)
Chronic fatigue (%)	9576 (67.5)
Restless legs syndrome (%)	2916 (20.6)
Extremity numbness (%)	8545 (60.2)
Fibromyalgia (%)	951 (6.7)
Interstitial cystitis (%)	790 (5.6)
Plantar fasciitis (%)	2183 (15.4)
PMS (%)	5099 (35.9)
Asthma (%)	737 (5.2)
Atopic dermatitis (%)	3754 (26.5)
Recurrent candidiasis (%)	2838 (20.0)
Chronic cervicalgia (%)	11,494 (81.0)

BMI, body mass index; PMS, premenstrual syndrome.

The mean IBS-SSS significantly improved after the intervention (before 287.3 ± 69.5 vs after 188.3 ± 87.6, *P* < .001). The intensity of GERD, dyspepsia, and belches was significantly reduced (*P* < .001). The intensity of all extraintestinal non-digestive symptoms was also significantly reduced (*P* < .001). The mean PHQ-9 score significantly improved from 10.2 ± 5.4 to 3.6 ± 3.4 (*P* < .001) (Table 2).

**Table 2:** Effect of the Dietary Intervention in Intestinal and Extraintestinal Symptoms

	Before intervention	After intervention	*P*
IBS symptoms			
Mean IBS-SSS score ±SD	287.3 ± 69.5	188.3 ± 87.6	**< .001**
Extraintestinal digestive symptoms (mean likert scale 0–10)			
GERD	4.6	1.3	**< .001**
Dyspepsia	4.3	1.4	**< .001**
Belches	4.6	2.0	**< .001**
Depressive symptoms			
Mean PHQ-9 score ±SD	10.2 ± 5.4	3.6 ± 3.4	**< .001**
Extraintestinal symptoms (mean likert scale 0–10)			
Migraine	6.1	3.9	**< .001**
Arthralgia	6.2	3.6	**< .001**
Chronic fatigue	6.4	3.8	**< .001**
Restless legs syndrome	5.5	2.9	**< .001**
Extremity numbness	5.4	3.1	**< .001**
Fibromyalgia	7.1	4.7	**< .001**
Interstitial cystitis	5.8	3.3	**< .001**
Plantar fasciitis	5.8	3.1	**< .001**
PMS	6.6	3.4	**< .001**
Asthma	4.2	2.5	**< .001**
Atopic dermatitis	5.7	3.1	**< .001**
Recurrent candidiasis	5.6	2.8	**< .001**
Chronic cervicalgia	6.8	3.9	**< .001**

*P* values <.05 are denoted in bold.

IBS-SSS, Irritable Bowel Syndrome Severity Scoring System; PMS, pre-menstrual syndrome; SD, standard deviation.

**Figure 2:**
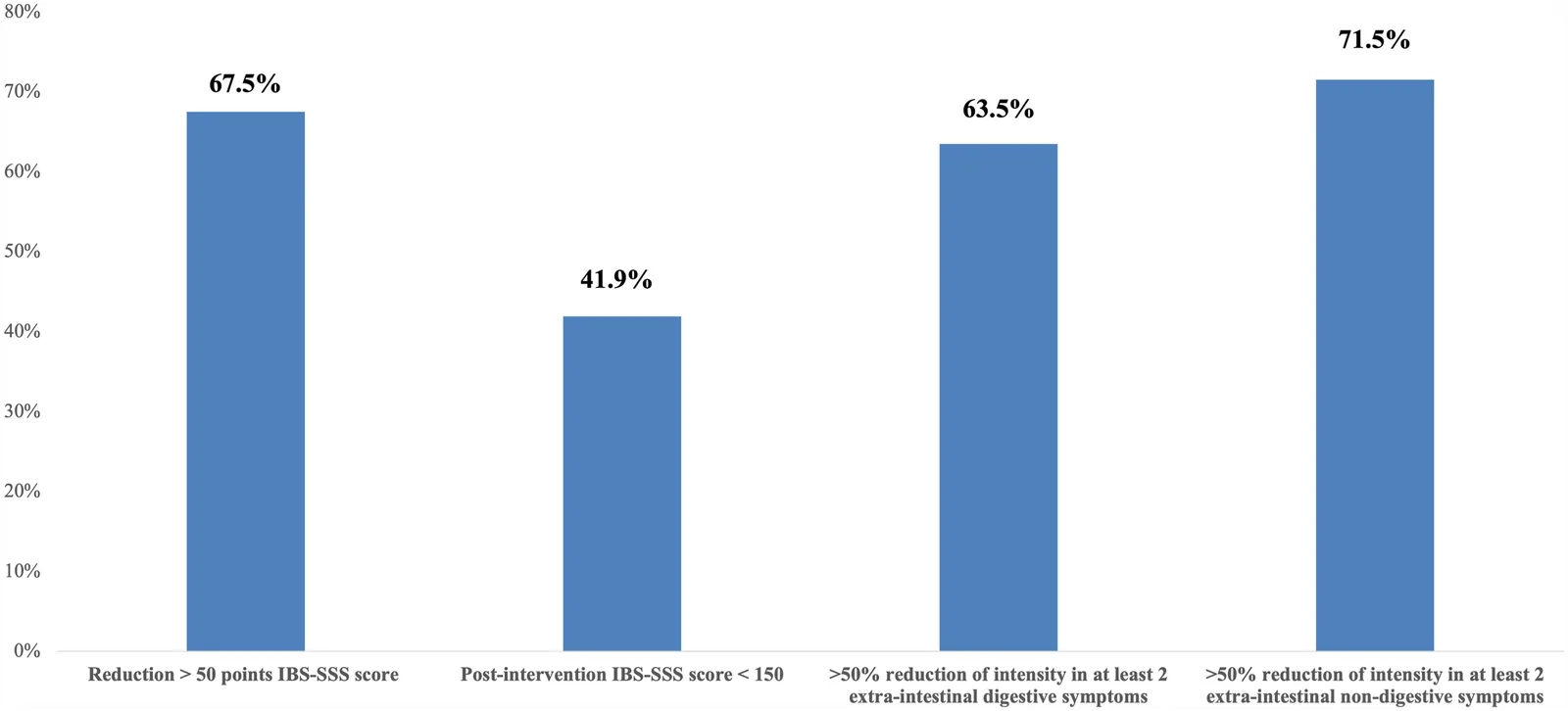
Rates of clinically meaningful improvement of IBS-related symptoms and extraintestinal digestive and non-digestive symptoms after the intervention.

Overall, 9576 (67.5%) showed a decrease of at least 50 points in the IBS-SSS score, and 5952 (41.9%) had a postintervention IBS-SSS score <150. In patients with multiple (2 or more) extraintestinal symptoms, 5540/8729 (63.5%) and 8403/11,746 (71.5%) reported >50% reduction of intensity in at least 2 extraintestinal digestive and non-digestive symptoms, respectively (Figure 2).

## Discussion

The aim of our study was to determine the impact of a holistic social media–based program on intestinal and extraintestinal symptoms in patients with IBS. We found that (a) extraintestinal and depressive symptoms are highly prevalent in these patients; (b) the intensity of IBS-related symptoms was significantly reduced after the intervention; and (c) depressive symptoms and extraintestinal digestive and non-digestive symptoms also improved significantly.

Previous studies have investigated the prevalence of extraintestinal and depressive symptoms in patients with IBS. Riedl and colleagues^18^ reported that these patients had a 2-fold increase in somatic comorbidities compared to controls, and fibromyalgia or chronic fatigue syndrome was documented in up to 65% of patients. A cross-sectional survey study from the Mayo Clinic showed that most IBS patients reported overlapping migraine (74%), depression (69%), anxiety (64%), and fibromyalgia (52%). In addition, patients with comorbid conditions were affected by higher symptom severity and had significantly lower quality of life.^19^ A previous meta-analysis assessed the prevalence of depression in adult patients with IBS and found that 28.8% of patients reported depressive symptoms and concluded that IBS was linked with 3-fold increased odds of depression.^20^ In line with these findings, our analysis showed that 85.5% of patients reported the presence of at least one extraintestinal symptom and almost 50% of patients had moderate-severe depressive symptoms, which highlights the need of a multidisciplinary therapy targeting a wide range of symptoms.

Most patients with IBS identify certain foods as a trigger for their symptoms. For this reason, dietary interventions which eliminate or restrict potential triggering foods have become the foundation of management of IBS.^21^ The low-FODMAPs diet, which limits wheat-based products, legumes, lactose, and certain vegetables (garlic, beans, onions) and fruits (blackberries, peaches), has been found superior to alternative dietary interventions.^22^ The diet is mainly effective for improving abdominal pain, distension, bloating, and bowel habits.^15^ In fact, a recent systemic review of randomized trials showed that the low-FODMAPs diet reduces the severity of gastrointestinal symptoms, mostly frequency and severity of abdominal pain and bloating. However, conflicting results were found regarding improvement of overall quality of life.^23^ In our large cohort of patients, we found significant reductions in IBS-SSS scores and improvement of gastrointestinal symptoms in almost 80% of patients after the dietary intervention. Furthermore, almost 70% of patients showed a decrease of at least 50 points in the IBS-SSS score after the intervention. Potential long-term effects such as alterations in composition of the gut microbiome and nutritional deficiencies should still be supervised and investigated in future studies.

A unique finding of our study was the effectiveness of our program in improving extraintestinal and depressive symptoms in most patients. The common pathophysiologic pathways of both gastrointestinal and extraintestinal symptoms (ie, visceral and somatic hypersensitivity) might partially explain the improvement of such a broad range of symptoms.^24^ For instance, previous research has shown strong interconnections between migraine and gastrointestinal symptoms due to alterations in the gut microbiota profile, diverse neuropeptide pathways, activation of inflammatory mediators, and nutritional deficiencies.^25^ We hypothesize that interventions beyond the dietary component included in our program (eg, exercise, yoga, mindfulness) also played a major role. Muscle relaxation exercises, physical activity, and yoga have demonstrated comprehensive benefits in patients with IBS, including reduction of symptom severity and improvement of quality of life and psychological well-being.^26^, ^27^, ^28^ Mindfulness has also shown to ameliorate gastrointestinal and extraintestinal symptoms in patients with IBS.^29^ Overall, our results and current evidence suggest that both dietary and non-dietary interventions are critical for the optimal management of this complex disease. Unfortunately, real-life implementation of comprehensive IBS treatment interventions is challenging due to limited available resources in most settings. For instance, broad availability of multidisciplinary teams (nutritionist coach, physician, physical trainer, etc.) capable of undergoing intensive monitoring of patients might not be possible. Therefore, the development of online accessible resources with reliable educational materials might help increasing access to effective therapeutic strategies for IBS.

Our study has several limitations that should be acknowledged. First, the analysis was based on self-reported data which is prone to recall, misclassification, and response bias. Second, as our study lacks longitudinal follow-up, we were unable to determine long-term symptomatic response. Third, no control group was included in the study, which precludes recognizing placebo effects or natural symptom fluctuation. Fourth, our cohort of patients from a single country might not be representative of the other populations with IBS with diverse dietary patterns and gut microbiome. Finally, information regarding concomitant medications and treatment modifications during the intervention was not systematically collected. Therefore, we cannot exclude that pharmacological therapies contributed, at least in part, to the observed improvements.

## Conclusion

A structured, coach-led, and peer-supported lifestyle program combining dietary guidance, behavioral support, and non-pharmacological strategies significantly improved both gastrointestinal and extraintestinal symptoms in a large cohort of patients with IBS. This integrative and scalable approach may help overcome the limitations of current therapies and address the broader symptom burden of IBS.

## References

[1] Lacy B.E., Mearin F., Chang L. (2016). Bowel disorders. Gastroenterology.

[2] Choi Y., Youn Y.H., Kang S.J. (2025). Korean Society of Neurogastroenterology and motility. 2025 Seoul Consensus on Clinical Practice guidelines for irritable bowel syndrome. J Neurogastroenterol Motil.

[3] Arif T.B., Ali S.H., Bhojwani K.D. (2025). Global prevalence and risk factors of irritable bowel syndrome from 2006 to 2024 using the Rome III and IV criteria: a meta-analysis. Eur J Gastroenterol Hepatol.

[4] Ohman L., Simrén M. (2010). Pathogenesis of IBS: role of inflammation, immunity and neuroimmune interactions. Nat Rev Gastroenterol Hepatol.

[5] Zhou Q., Fillingim R.B., Riley J.L. (2010). Central and peripheral hypersensitivity in the irritable bowel syndrome. Pain.

[6] Shiha M.G., Aziz I. (2021). Review article: physical and psychological comorbidities associated with irritable bowel syndrome. Aliment Pharmacol Ther.

[7] Ruderstam H., Ohlsson B. (2022). Self-reported IBS and gastrointestinal symptoms in the general population are associated with asthma, drug consumption and a family history of gastrointestinal diseases. Scand J Gastroenterol.

[8] Ohlsson B. (2022). Extraintestinal manifestations in irritable bowel syndrome: a systematic review. Therap Adv Gastroenterol.

[9] Memel Z.N., Shah N.D., Beck K.R. (2025). Diet, nutraceuticals, and lifestyle interventions for the treatment and management of irritable bowel syndrome. Nutr Clin Pract.

[10] Halmos E.P., Power V.A., Shepherd S.J. (2014). A diet low in FODMAPs reduces symptoms of irritable bowel syndrome. Gastroenterology.

[11] Staudacher H.M., Whelan K. (2017). The low FODMAP diet: recent advances in understanding its mechanisms and efficacy in IBS. Gut.

[12] Alrasheedi A.A., Jahlan E.A., Bakarman M.A. (2025). The effect of low-FODMAP diet on patients with irritable bowel syndrome. Sci Rep.

[13] Lacy B.E., Pimentel M., Brenner D.M. (2021). ACG clinical guideline: management of irritable bowel syndrome. Am J Gastroenterol.

[14] Manning L.P., Tuck C.J., Biesiekierski J.R. (2025). Predicting response to the low FODMAP diet in irritable bowel syndrome: current evidence and clinical considerations. Asia Pac J Clin Nutr.

[15] Jacobs J.P., Labus J.S., Dong T.S., Shin A.S., Mayer E.A., Chang L., UCLA SCORE Group in IBS and Sex Differences (2026). Extraintestinal symptoms in irritable bowel syndrome are associated with stress reactivity and the gut microbiome in a sex-dependent manner. Clin Gastroenterol Hepatol.

[16] Almansa C., García-Sánchez R., Barceló M. (2011). Translation, cultural adaptation and validation of a Spanish version of the Irritable Bowel Syndrome Severity score. Rev Esp Enferm Dig.

[17] Snijkers J.T.W., van den Oever W., Weerts Z.Z.R.M. (2021). Examining the optimal cutoff values of HADS, PHQ-9 and GAD-7 as screening instruments for depression and anxiety in irritable bowel syndrome. Neurogastroenterol Motil.

[18] Riedl A., Schmidtmann M., Stengel A. (2008). Somatic comorbidities of irritable bowel syndrome: a systematic analysis. J Psychosom Res.

[19] Wang X.J., Ebbert J.O., Loftus C.G. (2023). Comorbid extra-intestinal central sensitization conditions worsen irritable bowel syndrome in primary care patients. Neurogastroenterol Motil.

[20] Zamani M., Alizadeh-Tabari S., Zamani V. (2019). Systematic review with meta-analysis: the prevalence of anxiety and depression in patients with irritable bowel syndrome. Aliment Pharmacol Ther.

[21] Borghini R., Donato G., Alvaro D. (2017). New insights in IBS-like disorders: pandora's box has been opened; a review. Gastroenterol Hepatol Bed Bench.

[22] De Palma G., Bercik P. (2022). Long-term personalized low FODMAP diet in IBS. Neurogastroenterol Motil.

[23] Kuźmin L., Kubiak K., Lange E. (2025). Efficacy of a Low-FODMAP diet on the severity of gastrointestinal symptoms and quality of life in the treatment of gastrointestinal disorders-a systematic review of randomized controlled trials. Nutrients.

[24] Alpers D.H. (2008). Multidimensionality of symptom complexes in irritable bowel syndrome and other functional gastrointestinal disorders. J Psychosom Res.

[25] Arzani M., Jahromi S.R., Ghorbani Z. (2020). Gut-brain axis and migraine headache: a comprehensive review. J Headache Pain.

[26] Chao W.C., Huang J.C., Young S.L. (2024). Interplay of yoga, physical activity, and probiotics in irritable bowel syndrome management: a double-blind randomized study. Complement Ther Clin Pract.

[27] Arslan E., Kiyak E. (2025). The effects of progressive muscle relaxation exercises on symptom severity and quality of life in patients with irritable bowel syndrome. Gastroenterol Nurs.

[28] Lindsell H.B., Williams N.C., Magistro D. (2025). Could the therapeutic effect of physical activity on irritable bowel syndrome be mediated through changes to the gut microbiome? A narrative and hypothesis generating review. Neurogastroenterol Motil.

[29] Aghamohammadi V., Rezakhani Moghadam H., Najafi E. (2025). Mindfulness is associated with the weight status, severity of disease, and severity of extra-intestinal symptoms among individuals with irritable bowel syndrome. Front Psychol.

